# Effect of TIMP2/TIMP3 genes on the risk of osteosarcoma in Zhejiang population

**DOI:** 10.1097/MD.0000000000024818

**Published:** 2021-03-19

**Authors:** Zhongwei Wu, Huali Chen, Liwei Pan, Weiyang Yu, Chao Lou, Jian Chen, Dengwei He

**Affiliations:** a Spinal Surgery Department, The Central Hospital of Lishui City; b Orthopaedics Department, Lishui City People's Hospital, Lishui; c Spinal Surgery Department, The Second Affiliated Hospital of Wenzhou Medical University, Wenzhou, Zhejiang, China.

**Keywords:** genetic polymorphism, osteosarcoma, *TIMP2*, *TIMP3*, Zhejiang populations

## Abstract

Osteosarcoma is a malignant tumor that develops from a mesenchymal cell line and is caused by gene–environment interactions. This study aimed to explore whether *TIMP2*/*TIMP3* polymorphisms influenced the osteosarcoma risk.

The expression of the *TIMP2* and *TIMP3* genes in osteosarcoma histiocytes was analyzed by immunohistochemistry. In this case-control study, which includes samples from 499 patients and 500 healthy controls, 10 single-nucleotide polymorphisms (SNPs) in *TIMP2* and *TIMP3* were selected. Furthermore, we used the Agena MassARRAY platform for genotyping. The statistical analysis was performed using *χ*^2^ test/Fisher exact test, and logistic regression analysis.

The immunohistochemistry results showed that the expression of *TIMP2* is obvious higher in osteosarcoma histiocytes than in the normal histiocytes. The association study indicated that the allele of rs2277698 and rs4789936 were protective SNPs reducing the risk of osteosarcoma (odds ratios  > 1, *P* < .05) by the *χ*^2^ test. In the genetic model, logistic regression analyses revealed that the rs2277698 and rs4789936 were associated with decreasing the risk of osteosarcoma under the codominant model, dominant model, and log-additive model. Stratification analysis revealed that 2 SNPs (rs2277698 and rs4789936) were significantly associated with a reduced risk of osteosarcoma in allele and genetic model after stratification by gender or age (*P* < .05). In addition, the haplotype “T_rs2277698_C_rs2009169_C_rs7342880_” of *TIMP2* was associated with decreasing the osteosarcoma risk. The “A_rs9609634_T_rs11547635_” of *TIMP3* was associated with reducing the osteosarcoma risk.

This finding shed new light on the high expression of *TIMP2* polymorphisms may contribute to decreasing the osteosarcoma risk in Zhejiang populations.

## 1 Introduction

Osteosarcoma, one of the most common primary bone tumors, is highly aggressive and easily metastasizes which mainly occurs in teenagers and young adults.^[1]^ It develops from the mesenchymal cell line.^[2]^ The tumor grows rapidly and its prognosis is generally poor, accompanied by high mortality. Annual morbidity rate of osteosarcoma is about 0.3 to 0.5 per 10 million people across the world, and it presents a bimodal age distribution with peaks at 15 to 19 years old and 70 years old.^[3]^ The estimated 5-year survival rate of patients with distal metastasis is less than 30%, which makes osteosarcoma a severe 50 threat to young patients.^[4,5]^ It is known to all that osteosarcoma is complex and multifactorial disease, and the carcinogenesis of those malignant bone tumors is still uncertain.^[6]^

At present, a lot of research has been reported that there are gene–environment interactions in the carcinogenesis of malignant bone tumors.^[7,8]^ However, under the same risk factors, the onset of different individuals is different, which suggests that individual genetic background may play an essential role in determining the development of osteosarcoma.^[9]^ And this genetic background differences in the population mainly manifested as the single-nucleotide polymorphism (SNP). Therefore, the genetic susceptibility factors play a vital role in the development of osteosarcoma. Previously, genetic linkage analysis and candidate gene association studies in osteosarcoma have implicated several loci and candidate genes, for example, several study showed that the X-ray repair cross-complementing group-1 (*XRCC1*),^[10]^ excision repair cross-complementation (*ERCC*),^[10,11]^ 5,10-methylenetrahydrofolate reductase (*MTHFR*),^[12]^ insulin-like growth factor 1 (*IGF-1*),^[13]^ the apurinic/apyrimidinic endonuclease (*APE1*),^[14]^ and tumor suppressor gene *TP53*^[15]^ were associated with susceptibility to osteosarcoma.

The tissue inhibitors of metalloproteinases (TIMPs) including *TIMP2* and *TIMP3* are the key physiological inhibitors of matrix metalloproteinases (MMPs) and along with MMPs, TIMPs play a vital role in the basement membrane that represent the barriers to any malignant tumor invasion and progression.^[16]^ Many studies have reported *TIMP2* and *TIMP3* may be risk factors developing complex diseases,^[17]^ including colorectal cancer,^[16]^ urinary bladder cancer,^[18]^ coronary artery disease and myocardial infarction,^[19]^ and lumbar disc degeneration.^[20]^ However, few studies investigated the association of the *TIMP2* and *TIMP3* genes susceptibility to the osteosarcoma. Therefore, we performed a case-control study to analyze the association between the *TIMP2* and *TIMP3* genes and the risk of osteosarcoma from the teenagers in Zhejiang Province.

## 2 Materials and methods

### 2.1 Subject recruitment and ethics committee statement

We performed a case-control study to determine the association between *TIMP2*/*TIMP3* polymorphisms and osteosarcoma risk. A total of 499 osteosarcoma cases, and 500 controls were recruited from The Central Hospital of Lishui City between January 2016 and January 2019. Detailed recruitment and exclusion criteria were used. All the osteosarcoma cases were newly diagnosed and histologically confirmed. Patients who had any previous history of other cancers and who had undergone radiotherapy or chemotherapy before surgery were excluded. Control subjects were randomly selected from the medical examination center at the same hospital during the similar period.

All participants were informed both in writing and verbally of the procedures and purpose of the study, and they signed informed consent documents. The use of human tissue and the protocol in this study were strictly conformed to the principles expressed in the Declaration of Helsinki, and this study was carried out with approval from the ethics committee of The Central Hospital of Lishui City. All the subsequent research analyses were carried out in accordance with the approved guidelines and regulations.

### 2.2 Immunohistochemical (IHC) evaluation

The expression of *TIMP2* in the osteosarcoma tissue was also detected using immunohistochemistry. Specimens obtained from surgical resection were fixed in 10% formalin prior to being processed in paraffin. Immunohistochemical staining was performed using an EnVision TM HRP-polymer anti-mouse IHC Kit (K8002; Dake BioTECH, Shenzhen, China) according to the manufacturer's guidelines. The sections were stained within 5 days of cutting using an Autostainer Link48 (Dako, California, USA) in strict accordance with the manufacturer's instructions. The primary antibodies specific for TIMP2 (mouse TIMP2 (sc-21,735; Santa Cruz Biotechnology, Santa Cruz, CA), diluted 1:50) were obtained from Sigma-Aldrich (St. Louis, MO). Finally, we observed the images of the scanned tissue slices through Aperio ImageScope (Version 11.1.2.752).

### 2.3 SNP selection and genotyping

A GoldMag–Mini Purification Kit (GoldMag Co Ltd, Xian City, China) was used to extract genomic DNA from whole-blood samples. DNA samples were stored at − 20°C prior to analysis. At the same time, the concentrations and purity of the DNA were measured by using the NanoDrop 2000 (Thermo Fisher Scientific, Waltham, MA) at a wavelength of A260 and A280 nm.

Ten tag SNPs in *TIMP2* and *TIMP3* were selected for our study. These SNPs had minor allele frequencies greater than 5% according to the 1000 Genomes Project (http://www.internationalgenome.org/). The primers were designed online (https://agenacx.com/online-tools/). Agena MassARRAY Assay Design 4.0 software was used to design a multiplexed SNP MassEXTEND assay, and SNP genotyping was performed using the Agena MassARRAY RS1000 with manufacturer protocols. Agena Typer 4.0 software was used to perform data management and analyses.

### 2.4 Statistical analysis

Data analysis was performed using Microsoft Excel (Redmond, WA) and SPSS 19.0 statistical package (SPSS, Chicago, IL). Each SNP frequency in the control subjects was assessed for departure from Hardy–Weinberg Equilibrium (HWE) using an exact test. We calculated genotype frequencies of cases and controls using a *χ*^2^test. Odds ratios (ORs) and 95% confidence intervals (CIs) were determined using unconditional logistic regression with adjustment for age and sex. Five genetic models (codominant, dominant, recessive, and additive) were performed using PLINK software (http://zzz.bwh.harvard.edu/plink/anal.shtml), to characterize the potential association of *TIMP2*/*TIMP3* polymorphisms and osteosarcoma risk. Finally, we used Haploview software package (version 4.2) to evaluate pairwise linkage disequilibrium (LD), haplotype construction, and genetic association of the polymorphic loci. All *P* values were 2-sided, and *P* < .05 was indicated statistical significance.

## 3 Result

### 3.1 The expression of TIMP2/TIMP3 in the primary osteosarcoma histiocytes

**Figure 1:**
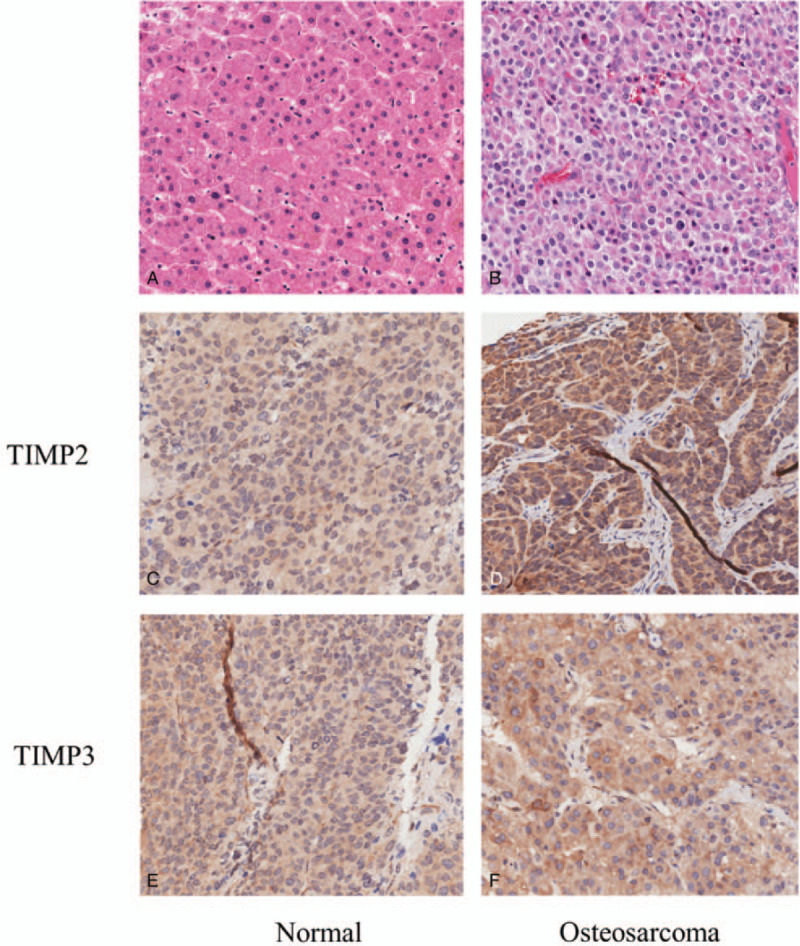
Morphological observation of normal histiocytes (A) and osteosarcoma histiocytes (B), and the expression of *TIMP2*/*TIMP3* in normal normal histiocytes (C, E) and osteosarcoma histiocytes (D, F). TIMPs = the tissue inhibitors of metalloproteinases.

As shown in Figure 1, we observed the morphological observation of normal histiocytes and osteosarcoma histiocytes by hematoxylin–eosin staining showed that there are obvious differences in morphology between osteosarcoma histiocytes and normal histiocytes under the electron microscope (×20), and the size and shape of osteosarcoma histiocytes are inconsistent, and the volume of nucleus increased (Fig. 1, A and B). Representative photomicrographs of staining intensity of *TIMP2* and *TIMP3* expressions in osteosarcoma histiocytes and normal histiocytes are shown in Figure 1C to F. Compared with Figure 1C, *TIMP2* expression was obviously enhanced in osteosarcoma histiocytes (Fig. 1D). However, there was no significant difference in the expression of *TIMP3* between osteosarcoma histiocytes (Fig. 1E) and normal histiocytes (Fig. 1F).

### 3.2 Characteristics of the participants

This study involved 999 subjects, including 499 patients (321 males and 178 females) and 500 healthy subjects (297 males and 203 females). The mean ages of teenagers were 15.12 ± 4.26 years for patients and 15.61 ± 5.73 years for controls. The mean ages of old peoples were 66.34 ± 3.76 years for patients and 67.08 ± 5.32 years for controls. The cases and controls were matched by age and sex, and there were no significant differences in the distributions of age and sex between osteosarcoma patients and healthy controls (*P *> .05) (Table 1).

**Table 1:** The characteristic of case and control.

Variable	Case	%	Control	%	*P*
Total	499		500		
Gender					>.05^∗^
Male	321	64.3	297	59.4	
Female	178	35.7	203	40.6	
Teenagers Age (yr, SD)	15.12 ± 4.26		15.61 ± 5.73		>.05^†^
Age≤24	386	77.3	221	44.2	
Old people Age (yr, SD)	66.34 ± 3.76		67.08 ± 5.32		>.05^†^
Age> 56	112	22.7	279	55.8	
Clinical stages					
Stage II	194	38.9			
Stage III	122	24.4			
Stage IV	183	36.7			

∗ *P* values were calculated from 2-sided *χ*^2^ tests.

† *P* values were calculated by Student *t* tests.

### 3.3 Associations between TIMP2 and TIMP3 SNPs and osteosarcoma risk

Ten SNPs in *TIMP2 and TIMP3* were analyzed in this study. Allele frequencies and basic information for all SNPs are shown in Table 2. All SNPs were in HWE in the controls (*P* > .05). We used the *χ*^2^ test to assess the risk of gene polymorphisms in the allele model, the frequency of the “T” allele of rs2277698 was significantly lower in cases than in controls (32.7% vs 33.3%), which suggested that “T” allele of rs2277698 was associated with decreasing the risk of osteosarcoma (OR = 0.29, 95% CI = 0.16–0.73, *P* = .015). The frequency of the “T” allele of rs4789936 was significantly lower in cases than in controls (22.1% vs 28.3%), which suggested that “T” allele of rs4789936 was a risk allele reducing the development of osteosarcoma (OR = 0.32, 95% CI = 0.17–0.884, *P* = .0014).

**Table 2:** Basic information of candidate SNPs and minor allele frequency between cases and controls.

				MAF				
SNPs	Locus	Gene(s)	Alleles A/B	Case	Control	HWE-*p*	OR (95% CI)	*P*^a^-values
rs2277698	17q25.3	*TIMP2*	T/C	0.327	0.333	0.763	0.29 (0.16–0.73)	.015
rs2009196	17q25.3	*TIMP2*	C/G	0.218	0.271	0.096	0.48 (0.17–1.89)	.216
rs7342880	17q25.3	*TIMP2*	A/C	0.227	0.300	0.193	1.14 (0.93–1.38)	.753
rs11654470	17q25.3	*TIMP2*	C/T	0.212	0.251	0.277	0.33 (0.24–1.70)	.614
rs2003241	17q25.3	*TIMP2*	C/T	0.116	0.131	0.134	0.46 (0.28–2.92)	.142
rs4789936	17q25.3	*TIMP2*	T/C	0.221	0.283	1.000	0.32 (0.17–0.88)	.0014
rs715572	22q12.3	*TIMP3*	A/G	0.102	0.119	0.579	1.02 (0.81–1.28)	.864
rs8136803	22q12.3	*TIMP3*	T/G	0.302	0.319	0.777	0.96 (0.79–1.18)	.721
rs9609643	22q12.3	*TIMP3*	A/G	0.058	0.058	0.226	0.62 (0.31–1.84)	.135
rs11547635	22q12.3	*TIMP3*	T/C	0.131	0.129	0.861	1.04 (0.83–2.31)	.323

Alleles A/B = Minor/major alleles, CI = confidence interval, HWE = Hardy–Weinberg equilibrium, MAF = minor allele frequency, OR = odds ratio, SNP = single-nucleotide polymorphism. *P* values were calculated using 2-sided *χ*^2^ test. *P *< .05 indicates statistical significance.

Furthermore, we assumed that the minor allele of each SNP as a risk factor compared with the wild-type allele. Four genetic models (codominant, dominant, recessive, and additive) were applied to analyze the associations between the SNPs and osteosarcoma risk using a logistic regression test. Our analyses showed that the rs2277698 in the *TIMP2* was associated with a 0.64-fold decreased the osteosarcoma risk under the co-dominant model (OR = 064, 95% CI = 0.43–0.83, *P* = .012 for the “T/T” genotype), 0.56-fold decreased the osteosarcoma risk under the dominant model (OR = 0.56, 95% CI = 0.21–0.92, *P* = .004 for the “C/T-T/T” genotype), and 0.36-fold decreased the osteosarcoma risk under the Log-additive model (OR = 0.36, 95% CI = 0.29–0.89, *P* = 0.039), respectively. The rs4789936 was associated with a 0.62-fold decreased the osteosarcoma risk under the codominant model (OR = 0.62, 95% CI = 0.25–0.91, *P* = .034 for the “T/T” genotype), 1.34-fold decreased the osteosarcoma risk under the dominant model (OR = 0.65, 95% CI = 0.42–0.97, *P* = .041 for the “C/T-T/T” genotype) and 1.46-fold decreased the risk of osteosarcoma under the Log-additive model (OR = 0.72, 95% CI = 0.51–0.95, *P* = .023), respectively (Table 3).

**Table 3:** Association between candidate SNPs and the risk of osteosarcoma under in genetic models.

SNPs	Models	Genotype	Control	Case	OR (95% CI)	*P* value	AIC	BIC
rs2277698	Codominant	C/C	217	183	1	**.012**	519.8	540.9
(*TIMP2*)		C/T	237	258	0.75 (0.66–1.63)			
		T/T	46	59	**0.64 (0.43–0.83)**			
	Dominant	C/C	217	183	1	**.004**	517.8	534.7
		C/T-T/T	283	317	**0.56 (0.21–0.92)**			
	Recessive	C/C-C/T	454	441	1	.960	517.9	534.7
		T/T	46	59	0.84 (0.43–2.43)			
	Log-additive	–	–	–	**0.36 (0.29–0.89)**	**.039**	517.8	534.7
rs2009196	Codominant	C/C	300	398	1	.122	516.7	537.7
		C/G	178	106	0.75 (0.45–1.26)			
		G/G	29	3	1.19 (0.65–2.18)			
	Dominant	C/C	300	398	1	.456	517.6	534.4
		C/G-G/G	207	109	0.88 (0.54–1.42)			
	Recessive	C/C-C/G	478	504	1	.416	515.8	532.7
		G/G	29	3	1.43 (0.87–2.37)			
	Log-additive	–	–	–	1.08 (0.80–1.46)	.160	517.6	534.4
rs7342880	Codominant	C/C	116	266	1	.331	519.4	540.4
		C/A	268	196	1.12 (0.71–1.76)			
		A/A	123	37	0.34 (0.07–1.74)			
	Dominant	C/C	116	266	1	.154	519.6	536.4
		C/A-A/A	391	232	1.05 (0.67–1.63)			
	Recessive	C/C-C/A	384	461	1	.216	517.6	534.4
		A/A	123	37	0.33 (0.06–1.66)			
	Log-additive	–	–	–	0.96 (0.64–1.43)	.284	519.6	536.4
rs11654470	Codominant	T/T	209	124	1	.168	520.8	541.8
		T/C	239	257	0.96 (0.61–1.51)			
		C/C	59	118	1.02 (0.48–2.14)			
	Dominant	T/T	209	124	1	.193	518.8	535.6
		T/C-C/C	298	375	0.97 (0.63–1.50)			
	Recessive	T/T-T/C	448	381	1	.192	518.8	535.6
		C/C	59	124	1.03 (0.51–2.10)			
	Log-additive	–	–	–	0.99 (0.71–1.37)	.296	518.8	535.6
rs2003241	Codominant	T/T	327	203	1	.463	514.5	535.5
		T/C	154	248	0.80 (0.51–1.26)			
		C/C	18	48	0.97 (0.36–2.59)			
	Dominant	T/T	327	203	1	.437	512.6	529.4
		T/C-C/C	172	296	0.82 (0.53–1.27)			
	Recessive	T/T-T/C	481	451	1	.195	513.4	530.2
		C/C	18	48	1.06 (0.40–2.79)			
	Log-additive	–	–	–	0.88 (0.61–1.26)	.491	512.9	529.8
rs4789936	Codominant	C/C	260	209	1	**.034**	515.7	536.7
		C/T	197	236	0.65 (0.42–1.96)			
		T/T	43	55	**0.62 (0.25–0.91)**			
	Dominant	C/C	260	209	1	**.041**	513.7	530.5
		C/T-T/T	240	301	**0.65 (0.42–0.97)**			
	Recessive	C/C-C/T	457	445	1	.500	517.1	533.9
		T/T	43	55	0.74 (0.31–1.77)			
	Log-additive	–	–	–	**0.72 (0.51–0.95)**	**.023**	514.1	530.9
rs715572	Codominant	G/G	227	316	1	.265	518.7	539.8
(*TIMP3*)		G/A	248	172	1.18 (0.75–1.87)			
		A/A	25	19	0.88 (0.43–1.80)			
	Dominant	G/G	227	316	1	.163	517.3	534.2
		G/A-A/A	273	191	1.11 (0.72–1.71)			
	Recessive	G/G-G/A	475	488)	1	.257	517.2	534.1
		A/A	25	19	0.82 (0.42–1.62)			
	Log-additive	–	–	–	1.01 (0.74–1.39)	.193	517.6	534.4
rs8136803	Codominant	G/G	205	179	1	.331	519.3	540.3
		G/T	231	237	0.59 (0.29–1.19)			
		T/T	63	83	0.00 (0.00-NA)			
	Dominant	G/G	205	179	1	.314	517.4	534.2
		G/T-T/T	294	320	0.59 (0.29–1.18)			
	Recessive	G/G-G/T	436	416	1	.269	519.5	536.3
		T/T	63	83	0.00 (0.00-NA)			
	Log-additive	–	–	–	0.59 (0.29–1.18)	.113	517.3	534.2
rs9609643	Codominant	G/G	241	248	1	.279	521	542
		G/A	203	191	0.95 (0.57–1.57)			
		A/A	56	60	1.86 (0.28–12.48)			
	Dominant	G/G	241	251	1	.394	519.4	536.3
		G/A-A/A	259	151	0.98 (0.60–1.61)			
	Recessive	G/G-G/A	444	439	1	.251	519	535.8
		A/A	56	60	1.88 (0.28–12.58)			
	Log-additive	–	–	–	1.02 (0.65–1.61)	.193	519.4	536.2
rs11547635	Codominant	C/C	278	218	1	.188	517.7	538.8
		T/C	164	231	1.06 (0.68–1.67)			
		T/T	58	50	1.22 (0.56–2.66)			
	Dominant	C/C	278	218	1	.171	515.9	532.7
		T/C-T/T	222	281	1.09 (0.71–1.67)			
	Recessive	C/C-T/C	442	449	1	.166	515.8	532.6
		T/T	58	50	1.18 (0.56–2.49)			
	Log-additive	–	–	–	1.09 (0.78–1.52)	.162	515.8	532.6

AIC = Akaike's Information criterion, BIC = Bayesian Information criterion, CI = confidence interval, OR = odds ratios. *P* values were calculated from Wald test adjusted for age and sex. *P *< .05 indicates statistical significance.

### 3.4 LD and haplotype association analysis

**Figure 2:**
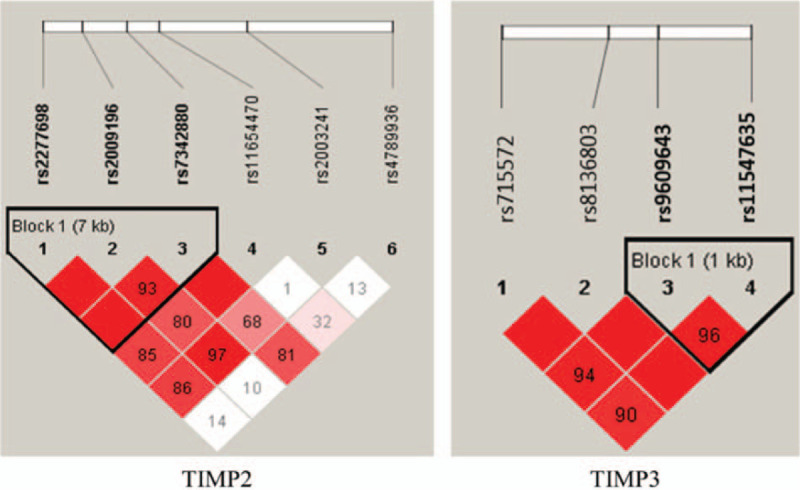
Haplotype block map for the *TIMP2* and *TIMP3* SNPs genotype in this study. SNP = single-nucleotide polymorphism.

Linkage disequilibrium and haplotype analyses of the SNPs in the case and control samples were further studied. Linkage disequilibrium structure is shown in Figure 2. We observed that the SNPs rs2277698, rs2009169, and rs7342880 in the *TIMP1* had very strong linkage disequilibria, it forms one LD block. One block was detected in studied *TIMP2* SNPs (rs9609643 and rs11547635) by haplotype analyses.

The haplotypes of the different blocks of each gene were calculated as shown in Table 4. The most frequent haplotype was used as reference, haplotype analysis of genes *TIMP2* and *TIMP3* detected significant association with the risk of osteosarcoma. The result showed that the “TCC” haplotype in the *TIMP2* (consisted of rs2277698, rs2009169, and rs7342880) was associated with decreasing the osteosarcoma risk (OR = 0.66, 95% CI: 0.48–0.96, *P* = .031). The “AT” haplotype in the *TIMP3* (consisted of rs9609634 and rs11547635) was associated with decreasing the osteosarcoma risk (OR = 0.64, 95% CI: 0.43–0.91, *P* = .046).

**Table 4:** Haplotype analysis results in this study.

Chromosome	Gene	SNPs	Haplotype	OR (5% CI)	*P* values
chr17	*TIMP2*	rs2277698|rs2009169|rs7342880	CGC	1	–
			TCC	**0.66 (0.48–0.96)**	**.031**
			CCA	0.90 (0.58–1.38)	.620
			CCC	0.76 (0.43–1.34)	.350
chr22	*TIMP3*	rs9609643|rs11547635	GC	1	.631
			AT	**0.64 (0.43–0.91)**	**.046**
			GT	0.89 (0.59–1.36)	.189

CI = confidence interval, OR = odds ratio, SNP = single-nucleotide polymorphism. *P* indicates adjusted by gender and age. *P* < .05 indicates statistical significance.

### 3.5 Stratification analysis

As shown in Table 5, we implemented a stratification analysis by gender and age to evaluate sex and age-specific associations between SNP alleles and osteosarcoma risk. In the allele model, we found that rs2277698 (*TIMP2*) significantly reduced the risk of osteosarcoma in males (OR = 0.57, 95% confidence interval [95% CI] = 0.25–0.9, *P* = .006; OR = 0.35, 95% CI = 0.26–0.77, *P* = .029, females (OR = 0.52, 95% CI = 0.33–0.85, *P* = .041), people aged under 24 (OR = 0.43, 95% CI = 0.26–0.91, *P* = .037; OR = 32, 95%CI = 0.21–0.68, *P* = .028), and the population over 56 years of age (OR = 0.51, 95% CI = 0.24–0.76, *P* = .018; OR = 0.43, 95% CI = 0.23–0.81, *P* = .047). In addition, the rs4789936 were associated with a decreased risk of osteosarcoma in males (OR = 0.64, 95% CI = 0.21–0.97, *P* = .016; OR = 0.71, 95% CI = 0.52–0.96, *P* = .039), people aged under 24 (OR = 0.53, 95% CI = 0.23–0.86, *P* = .011; OR = 0.47, 95% CI = 0.26–0.83, *P* = .036), and the population over 56 years of age (OR = 0.68, 95% CI = 0.37–0.96, *P* = .044; OR = 0.52, 95% CI = 0.35–0.84, *P* = .021).

**Table 5:** The association between sex and age stratification and osteosarcoma risk in allele and genotype models.

		Male		Female		Age ≤24		Age≥56	
SNPs	Alleles	OR (95% CI)	*P*^a^	OR (95% CI)	*P*^a^	OR (95% CI)	*P*^b^	OR (95% CI)	*P*^b^
*TIMP2*									
rs2277698	C/C	1	.034	1	.041	1	.037	1	.018
	C/T	0.79 (0.53–1.28)		0.95 (0.77–1.44)		0.80 (0.74–1.59)		0.92 (0.88–1.90)	
	T/T	0.57 (0.25–0.92)		0.52 (0.33–0.85)		0.43 (0.26–0.91)		0.51 (0.24–0.76)	
	C	1	.029	1	.616	1	.028	1	.047
	T	0.35 (0.26–0.77)		1.49 (0.30–1.82)		0.32 (0.21–0.68)		0.43 (0.23–0.81)	
rs2009196	C/C	1	.123	1	.085	1	.211	1	.056
	C/G	0.54 (0.38–1.78)		0.83 (0.67–1.13)		0.71 (0.53–1.93)		0.77 (0.59–1.01)	
	G/G	0.68 (0.21–2.57)		1.15 (0.84–1.97)		0.62 (0.35–3.94)		1.03 (0.65–1.64)	.897
	C	1	.186	1	.266	1	.598	1	
	G	0.52 (0.82–2.83)		1.17 (0.67–2.40)		1.12 (0.73–1.72)		0.98 (0.69–1.76)	
rs7342880	C/C	1	.254	1	.471	1	.144	1	.784
	C/A	1.05 (0.53–1.77)		0.63 (0.54–1.99)		0.54 (0.37–1.79)		1.16 (0.79–1.71)	
	A/A	0.48 (0.16–1.58)		0.78 (0.44–1.40)		0.57 (0.29–1.15)		1.09 (0.48–2.52)	
	C	1	.357	1	.178	1	.601	1	.406
	A	0.64 (0.44–1.79)		0.94 (0.62–1.83)		0.89 (0.53–1.51)		0.85 (0.51–1.42)	
rs11654470	T/T	1	.517	1	.251	1	.876	1	.476
	T/C	1.01 (0.79–1.55)		0.97 (0.79–1.19)		0.98 (0.77–1.25)		0.90 (0.70–1.17)	
	C/C	0.89 (0.56–2.31)		1.21 (0.99–2.00)		0.75 (0.52–1.09)		1.01 (0.67–1.49)	
	T	1	.321	1	.266	1	.134	1	1.148
	C	0.73 (0.53–1.23)		0.95 (0.68–1.49)		0.92 (0.64–1.36)		0.97 (0.87–1.91)	
rs2003241	T/T	1	.342	1	.542	1	.198	1	.219
	T/C	1.26 (0.89–2.04)		1.19 (0.86–1.61)		1.07 (0.77–1.50)		1.02 (0.74–1.65)	
	C/C	0.77 (0.71–2.16)		1.24 (0.59–2.07)		1.20 (0.84–1.71)		0.98 (0.60–2.00)	
	T	1	.176	1	.149	1	.155	1	.416
	C	1.15 (0.94–1.84)		1.02 (0.64–1.86)		0.79 (0.57–1.19)		0.96 (0.72–1.84)	
rs4789936	C/C	1	.016	1	.069	1	.011	1	.044
	C/T	0.55 (0.37–1.88)		1.21 (0.84–1.99)		0.72 (0.35–1.84)		0.53 (0.38–1.06)	
	T/T	0.64 (0.21–0.97)		1.38 (0.99–2.05)		0.53 (0.23–0.86)		0.68 (0.37–0.96)	
	C	1	.039	1	.087	1	.036	1	.021
	T	0.71 (0.52–0.96)		1.34 (0.87–2.03)		0.47 (0.26–0.83)		0.52 (0.35–0.84)	
*TIMP3*									
rs715572	G/G	1	.337	1	.172	1	.241	1	.142
	G/A	0.88 (0.49–1.54)		1.16 (0.85–1.83)		1.25 (0.83–2.07)		1.04 (0.72–1.41)	
	A/A	1.09 (0.65–1.93)		1.09 (0.72–1.91)		1.30 (0.92–1.86)		1.13 (0.81–2.00)	
	G	1	.452	1	.093	1	.332	1	.119
	A	1.06 (0.74–1.39)		1.25 (0.61–1.58)		0.84 (0.61–1.73)		0.96 (0.55–1.89)	
rs8136803	G/G	1	.259	1	.275	1	.625	1	.551
	G/T	1.06 (0.79–1.80)		0.89 (0.61–2.12)		1.16 (0.94–2.06)		1.09 (0.89–1.64)	
	T/T	0.96 (0.73–1.99)		0.95 (0.64–1.84)		1.27 (0.77–2.06)		1.15 (0.81–1.89)	
	G	1	.517	1	.361	1	.286	1	.362
	T	0.63 (0.37–2.05)		1.09 (0.84–1.67)		1.51 (0.78–2.17)		1.25 (0.99–1.86)	
rs9609643	G/G	1	.142	1	.095	1	.177	1	.247
	G/A	1.21 (0.93–1.82)		0.88 (0.46–2.32)		1.23 (0.89–1.71)		1.08 (0.96–1.70)	
	A/A	0.98 (0.52–1.67)		0.83 (0.63–2.04)		1.08 (0.66–1.87)		0.96 (0.37–2.01)	
	G	1	.323	1	.197	1	.664	1	.156
	A	1.12 (0.86–1.91)		1.25 (0.84–1.93)		0.77 (0.61–1.38)		1.01 (0.59–1.62)	
rs11547635	C/C	1	.359	1	.089	1	.337	1	.352
	T/C	1.23 (0.96–1.54)		1.09 (0.97–1.82)		1.31 (0.94–2.15)		1.08 (0.73–1.99)	
	T/T	1.09 (0.89–1.96)		0.76 (0.44–2.01)		0.98 (0.69–2.35)		1.21 (0.94–1.68)	
	C	1	.065	1	.168	1	.671	1	.527
	T	1.26 (0.99–2.05)		0.81 (0.51–1.92)		1.06 (0.62–1.87)		1.36 (0.85–2.10)	

95% CI = 95% confidence interval, OR = odds ratio. *P*^*a*^-values were calculated from Wald test adjusted for age. *P*^*b*^-values were calculated from Wald test adjusted for gender. *P* < .05 indicates statistical significance.

After stratification by age and gender in the genetic model (Table 6), rs2277698 was significantly associated with a decreased risk of osteosarcoma in males (dominant model: OR = 0.69, 95% CI = 0.48–0.89, *P* = .019 for the “C/T-T/T” genotype; log-additive model: OR = 0.46, 95% CI = 0.38–0.72, *P* = .026), females (log-additive model: OR = 0.65, 95% CI = 0.36–0.89, *P* = .042), the population under 24 years of age (dominant model: OR = 0.66, 95% CI = 0.47–0.93, *P* = .031; log-additive model: OR = 0.72, 95% CI = 0.55–0.94, *P* = .029), and over 56 years of age (dominant model: OR = 0.62, 95% CI = 0.35–0.81, *P* = .036). Also, rs4789936 has a protective effect in reducing the risk of osteosarcoma in males (dominant model: OR = 0.58, 95% CI = 0.36–0.91, *P* = 0.029 for the “C/T-T/T” genotype; log-additive model: OR = 0.56, 95% CI = 0.33–0.94, *P* = .041), the population under 24 years of age (dominant model: OR = 0.67, 95% CI = 0.34–0.96, *P* = .011; log-additive model: OR = 0.66, 95% CI = 0.32–0.97, *P* = .042), and over 56 years of age (log-additive model: OR = 0.61, 95% CI = 0.49–0.88, *P* = .019).

**Table 6:** The association between sex and age stratification and osteosarcoma risk under genetic models.

			Male		Female		Age ≤24		Age≥56	
SNPs	Model	Genotype	OR (95% CI)	*P*^a^	OR (95% CI)	*P*^a^	OR (95% CI)	*P*^b^	OR (95% CI)	*P*^b^
*TIMP2*										
rs2277698	Dominant	C/C	1	.019	1	.085	1	.031	1	.036
		C/T-T/T	0.69 (0.48–0.89)		0.96 (0.69–1.34)		0.66 (0.47–0.93)		0.62 (0.35–0.81)	
	Recessive	C/C-C/T	1	.094	1	.176	1	.057	1	.145
		T/T	0.55 (0.29–1.03)		0.87 (0.62–1.23)		0.76 (0.53–1.08)		0.72 (0.38–1.37)	
	Log-additive	–	0.46 (0.38–0.72)	.026	0.65 (0.36–0.89)	.042	0.72 (0.55–0.94)	.029	0.91 (0.62–1.33)	.113
rs2009196	Dominant	C/C	1	.265	1	.226	1	.634	1	.155
		C/G-G/G	1.40 (0.97–2.02)		0.83 (0.58–1.20)		0.90 (0.48–1.69)		0.85 (0.60–1.20)	
	Recessive	C/C-C/G	1	.512	1	.317	1	.391	1	.237
		G/G	0.72 (0.22–2.32)		0.82 (0.64–1.06)		0.93 (0.66–1.32)		0.76 (0.42–1.35)	
	Log-additive	–	0.95 (0.53–1.68)	.224	0.90 (0.69–1.17)	.121	1.00 (0.46–2.15)	.312	0.90 (0.63–1.28)	.334
rs7342880	Dominant	C/C	1	.326	1	.283	1	.482	1	.241
		C/A-A/A	0.87 (0.58–1.31)		0.57 (0.32–1.01)		0.84 (0.60–1.19)		0.86 (0.62–1.23)	
	Recessive	C/C-C/A	1	.113	1	.534	1	.336	1	.223
		A/A	0.90 (0.55–1.47)		0.67 (0.39–1.16)		0.58 (0.33–1.00)		0.97 (0.53–1.79)	
	Log-additive	–	0.88 (0.60–1.30)	.134	0.81 (0.33–2.00)	.201	1.04 (0.49–2.22)	.307	0.91 (0.67–1.40)	.406
rs11654470	Dominant	T/T	1	.167	1	.261	1	.297	1	.116
		T/C-C/C	0.98 (0.65–1.49)		0.92 (0.65–1.29)		0.92 (0.65–1.29)		0.75 (0.51–1.12)	
	Recessive	T/T-T/C	1	.142	1	.288	1	.313	1	.531
		C/C	0.93 (0.32–2.69)		0.77 (0.52–1.12)		1.00 (0.35–2.88)		1.00 (0.35–2.88)	
	Log-additive	–	0.82 (0.59–1.14)	.216	1.08 (0.62–1.87)	.316	1.19 (0.84–1.68)	.301	0.98 (0.58–1.64)	.357
rs2003241	Dominant	T/T	1	.235	1	.159	1	.362	1	.311
		T/C-C/C	1.09 (0.85–1.40)		1.03 (0.69–1.54)		1.04 (0.82–1.32)		1.02 (0.71–1.47)	
	Recessive	T/T-T/C	1	.089	1	.342	1	.144	1	.187
		C/C	1.00 (0.71–1.42)		0.92 (0.64–1.32		0.84 (0.38–1.87)		0.91 (0.64–1.29)	
	Log-additive	–	0.91 (0.64–1.29)	.139	0.93 (0.66–1.33)	.203	0.92 (0.69–1.23)	.094	0.91 (0.63–1.30)	.108
rs4789936	Dominant	C/C	1	.029	1	.067	1	.011	1	.114
		C/T-T/T	0.58 (0.36–0.91)		0.78 (0.61–1.01)		0.67 (0.34–0.96)		0.83 (0.58–1.21)	
	Recessive	C/C-C/T	1	.082	1	.117	1	.099	1	.235
		T/T	0.95 (0.64–1.41)		0.69 (0.35–1.38)		0.83 (0.58–1.21)		0.95 (0.64–1.40)	
	Log-additive	–	0.56 (0.33–0.94)	.041	0.74 (0.52–1.07)	.104	0.66 (0.32–0.97)	.042	0.61 (0.49–0.88)	.019
*TIMP3*										
rs715572	Dominant	G/G	1	.096	1	.324	1	.119	1	.235
		G/A-A/A	0.94 (0.66–1.33)		0.90 (0.63–1.27)		0.99 (0.75–1.30)		0.94 (0.63–1.42)	
	Recessive	G/G-G/A	1	.198	1	.186	1	.231	1	.164
		A/A	0.96 (0.65–1.43)		0.94 (0.63–1.41)		0.98 (0.69–1.41)		0.81 (0.57–1.17)	
	Log-additive	–	1.22 (0.32–4.64)	.217	1.24 (0.33–4.69)	.109	1.18 (0.60–2.34)	.106	1.13 (0.56–2.28)	.235
rs8136803	Dominant	G/G	1	.075	1	.311	1	.246	1	.217
		G/T-T/T	0.60 (0.29–1.25)		0.78 (0.55–1.10)		0.65 (0.32–1.34)		0.86 (0.60–1.22)	
	Recessive	G/G-G/T	1	.342	1	.242	1	.337	1	.099
		T/T	0.79 (0.60–1.05)		0.99 (0.68–1.42)		0.96 (0.54–1.70)		0.98 (0.70–1.38)	
	Log-additive	–	0.96 (0.56–1.67)	.116	0.99 (0.70–1.41)	.236	0.98 (0.76–1.27)	.203	0.92 (0.51–1.67)	.113
rs9609643	Dominant	G/G	1	.196	1	.151	1	.193	1	.341
		G/A-A/A	1.00 (0.71–1.43)		0.97 (0.75–1.26)		0.94 (0.59–1.49)		0.91 (0.52–1.44)	
	Recessive	G/G-G/A	1	.185	1	.206	1	.175	1	.216
		A/A	0.93 (0.59–1.47)		0.91 (0.58–1.45)		0.89 (0.56–1.42)		1.01 (0.64–1.59)	
	Log-additive	–	0.97 (0.68–1.37)	.157	1.01 (0.78–1.30)	.153	1.05 (0.58–1.91)	.104	1.06 (0.60–1.89)	.224
rs11547635	Dominant	C/C	1	.239	1	.091	1	.081	1	.167
		T/C-T/T	0.91 (0.62–1.34)		0.96 (0.65–1.41)		0.85 (0.66–1.10)		0.99 (0.68–1.43)	
	Recessive	C/C-T/C	1	.138	1	.154	1	.094	1	.076
		T/T	0.95 (0.67–1.34)		0.83 (0.58–1.21)		0.93 (0.58–1.46)		0.78 (0.60–1.00)	
	Log-additive	–	1.04 (0.64–1.67)	.214	1.11 (0.86–1.42)	.113	0.87 (0.62–1.23)	.107	0.96 (0.69–1.34)	.116

95% CI = 95% confidence interval, OR = odds ratio. *P*^*a*^-values were calculated from Wald test adjusted for age. *P*^*b*^-values were calculated from Wald test adjusted for gender. *P* < .05 indicates statistical significance.

## 4 Discussion

Genetic studies have provided insight into many diseases, including osteosarcoma. In the present case–control study, we investigated the associations between 10 SNPs in *TIMP2* and *TIMP3* genes and osteosarcoma risk in Zhejiang population. Our results show that the rs2277698 and rs4789936 in the *TIMP2* were associated with decreasing the risk of osteosarcoma. These results suggested that the polymorphisms of *TIMP2* gene may contribute to be a protective role reducing the osteosarcoma risk. In addition, we first used IHC to detect the expression of the *TIMP2* and *TIMP3* gene in normal histiocytes and osteosarcoma histiocytes. We found that the expression level of *TIMP2* in osteosarcoma histiocytes was significantly higher than the normal histiocytes. We predicted that this gene may be a risky gene for osteosarcoma.

The *TIMP2* is located on the long arm of chromosome 17 at position 25.3 (17q25.3). However, in addition to the MMP inhibitory activities, *TIMPs* play essential roles in many physiological processes including modulation of cell proliferation, migration, and invasion and synaptic plasticity.^[21]^*TIMPs* influence tumor progression and metastasis through the inhibition of *MMPs* and through direct modulation of angiogenesis and apoptosis.^[21,22]^ Many studies have shown that *TIMP2*, as a disease susceptibility gene, can affect the development of cancers and other diseases. For examples, Mikołajczyk-Stecyna et al^[23]^ reported that *TIMP2* was associated with increasing the risk of abdominal aortic aneurysm in the Polish population. Banday and Sameer^[16]^ demonstrated that there was a strong and highly significant association between the *TIMP2*-418G/C promoter SNPs and the risk of developing CRC in ethnic Kashmiri population. An et al^[24]^ showed that the *TIMP2* G > C (rs8179090) and G > A (rs2277698) alleles were strongly associated with primary ovarian insufficiency (POI), which suggested that the minor *TIMP2* alleles may increase POI risk in Korean women. This study identified that the rs2277698 and rs4789936 in the *TIMP2* were associated with decreasing the risk of osteosarcoma in Zhejiang populations, and found the expression level of *TIMP2* in osteosarcoma histiocytes was significantly higher than the normal histiocytes.

Tissue inhibitor of metalloproteinase 3, a member of the *TIMP* family, is located on the long arm of chromosome 22 at position 12.3 (22q12.3), which functions as the antagonist of MMPs to guard homeostasis and affect physiological tissue remodeling and developmental processes by regulating cell growth, invasion, migration, apoptosis, and angiogenesis.^[22,25]^ Furthermore, genetic variation in *TIMP3* has been linked with susceptibility to cardiovascular disorders and cancers. Perera et al^[20]^ found that the rs9862 variant of the *TIMP3* gene was associated with severity of lumbar disc degeneration and modic changes. Srivastava et al^[26]^ reported that *TIMP3* gene was associated with reducing the risk of prostate cancer in North Indian cohort. Banday and Sameer^[16]^ demonstrated that the *TIMP3*-1296T/C promoter SNPs was associated with decreased risk of colorectal cancer in ethnic Kashmiri population. However, few previous studies have reported associations between *TIMP3* gene polymorphism and osteosarcoma risk. Moreover, there was no significant difference in the expression level of *TIMP3* between normal tissue and osteosarcoma tissue.

Our study aimed to report the association between the polymorphisms of *TIMP2* and *TIMP3* and the osteosarcoma risk in the Zhejiang teenagers, which may provide new data to facilitate earlier diagnosis and promote early prevention, and shed light on the new candidate genes and new ideas for the study of subsequent occurrence mechanism of osteosarcoma. However, some potential limitations of our current study should be considered when deciphering the results. Our study only is a preliminary basic research, further functional studies and larger population-based prospective studies are required to understand the genetic factors underlying osteosarcoma in the subsequent research.

## 5 Conclusion

The results indicate that the expression level of *TIMP2* in osteosarcoma histiocytes was significantly higher than the normal histiocytes. The polymorphisms of *TIMP2* (rs2277698 and rs4789936) were significantly associated with decreasing the osteosarcoma risk.

## Acknowledgments

The authors thank all the patients and individuals for their participation. The authors thank the physicians and nurses of the 3 hospitals for their offers of osteosarcoma blood samples.

## Author contributions

**Conceptualization:** Chao Lou.

**Data curation:** Liwei Pan, Jian Chen.

**Formal analysis:** Jian Chen.

**Investigation:** Weiyang Yu.

**Methodology:** Weiyang Yu.

**Project administration:** Dengwei He.

**Resources:** Weiyang Yu.

**Supervision:** Chao Lou, Dengwei He.

**Writing – original draft:** Zhongwei Wu, Huali Chen.

**Writing – review & editing:** Zhongwei Wu, Huali Chen.
